# Prolonged Remission of Major Depressive Disorder After Single Nitrous Oxide Inhalation Treatment

**DOI:** 10.3389/fpsyt.2020.00692

**Published:** 2020-07-17

**Authors:** Peter Nagele, Frank Brown, Victoria Bass, Daniel Yohanna

**Affiliations:** ^1^ Department of Anesthesia and Critical Care, University of Chicago, Chicago, IL, United States; ^2^ Department of Psychiatry and Behavioral Neuroscience, University of Chicago, Chicago, IL, United States

**Keywords:** depression, nitrous oxide, remission, psychopharmacology, NMDA receptor

Nitrous oxide (N_2_O, laughing gas) has shown early promise as a rapidly acting treatment for treatment-resistant major depression (1–3). Antidepressant effects of nitrous oxide in these studies started within 2 h and lasted up to 1 week. Due to the study design, however, it was impossible to determine whether antidepressant efficacy lasted beyond 1 week after nitrous oxide administration. There is currently no evidence showing how long antidepressant effects of nitrous oxide last. Here, we present a case where a patient with severe recurrent treatment resistant major depression experienced full remission, which lasted more than 1 month, after a single nitrous oxide inhalation treatment.

## Case Report

Mr. X is a 41-year-old, married male patient with a more than 15-year history of unipolar depression. His first episode occurred during graduate school, was unsuccessfully treated with cognitive behavioral therapy alone, and lasted for 1 year. Subsequently, fluoxetine was tried (initially 20 mg/day for 6 months then increased to 60 mg/day for 6 months) but failed, and so the patient was switched to bupropion (150 mg/d) which resulted in improvement and ultimate remission. The patient did well up to 1 year prior to evaluation for his current depression. The precipitant of his current depression was due to unresolved marital discourse. During that year marital therapy, cognitive behavioral therapy, and adequate antidepressant treatment (bupropion initially 300 mg/d for 6 months, then 450 mg/d) were ineffective to relieve his depression. The patient approached us whether a treatment with nitrous oxide could be considered. In mid-December 2019 in our initial evaluation, the patient was severely depressed (PHQ-9 [Patient Health Questionnaire]: 22; GAD-7 [Generalized Anxiety Disorder Scale): 14) and after excluding potential contraindications for nitrous oxide (such as chronic vitamin B_12_ deficiency, middle ear occlusion) (4) and providing informed consent, we treated the patient with 50% nitrous oxide (mixed with 50% oxygen) inhalation (Porter Sentry Sedate MXR-D, Porter Instrument Division, Parker Hannifin, Hatfield, PA) for 1 h under continuous standard monitoring conditions (pulse oximetry, non-invasive blood pressure, ECG, end-tidal CO_2_) with an attending anesthesiologist continuously present. The patient experienced the treatment without any adverse events and recovered within a few minutes after cessation of gas administration. Within half an hour, the patient showed improved symptoms (smiling, whistling); on the next day, the patient reported reduced fear, increased joy, and overall improved symptoms. A month later (end of January 2020), the patient reported a PHQ-9 score of 0, indicating full remission, and again in mid-February he reported a PHQ-9 score of 0. We prescribed mirtazapine 15 mg for depression, poor sleep, and poor appetite, but due to the rapid improvement in symptoms, he decided not to take it.

While only a single case report, it represents evidence that a single 1-h inhalation treatment with 50% nitrous oxide may improve and even remit major depressive disorder for more than a month (4). The mechanism of nitrous oxide’s antidepressant effect is poorly understood and it presumed to involve NMDA-receptor antagonism (5).

## Ethics Statement

Written informed consent was obtained from the individual(s) for the publication of any potentially identifiable images or data included in this article.

## Author Contributions

PN drafted the report. FB, VB, and DY edited the report and made important intellectual contributions to the draft.

## Conflict of Interest

PN is currently receiving funding from NIMH, American Foundation for Prevention of Suicide, Brain Behavior Foundation, and has received research funding and honoraria from Roche Diagnostics and Abbott Diagnostics.

The remaining authors declare that the research was conducted in the absence of any commercial or financial relationships that could be construed as a potential conflict of interest.
